# Maintenance of bacterial outer membrane lipid asymmetry: insight into MlaA

**DOI:** 10.1186/s12866-023-03138-8

**Published:** 2024-05-28

**Authors:** M. Kaur, M.-P. Mingeot -Leclercq

**Affiliations:** Louvain Drug Research Institute, Université catholique de Louvain, Unité de Pharmacologie cellulaire et moléculaire, B1.73.05; 73 Av E. Mounier, Brussels, 1200 Belgium

**Keywords:** Outer membrane asymmetry, Gram-negative, MlaA, Phospholipids, Mla system

## Abstract

The outer membrane (OM) of Gram-negative bacteria acts as an effective barrier to protect against toxic compounds. By nature, the OM is asymmetric with the highly packed lipopolysaccharide (LPS) at the outer leaflet and glycerophospholipids at the inner leaflet. OM asymmetry is maintained by the Mla system, in which is responsible for the retrograde transport of glycerophospholipids from the OM to the inner membrane. This system is comprised of six Mla proteins, including MlaA, an OM lipoprotein involved in the removal of glycerophospholipids that are mis-localized at the outer leaflet of the OM. Interestingly, MlaA was initially identified - and called VacJ - based on its role in the intracellular spreading of *Shigella flexneri*.

Many open questions remain with respect to the Mla system and the mechanism involved in the translocation of mislocated glycerophospholipids at the outer leaflet of the OM, by MlaA. After summarizing the current knowledge on MlaA, we focus on the impact of *mlaA* deletion on OM lipid composition and biophysical properties of the OM. How changes in OM lipid composition and biophysical properties can impact the generation of membrane vesicles and membrane permeability is discussed. Finally, we explore whether and how MlaA might be a candidate for improving the activity of antibiotics and as a vaccine candidate.

Efforts dedicated to understanding the relationship between the OM lipid composition and the mechanical strength of the bacterial envelope and, in turn, how such properties act against external stress, are needed for the design of new targets or drugs for Gram-negative infections.

## Gram-negative bacteria: outer membrane (OM) asymmetry

The cell envelope of Gram-negative bacteria is important for maintaining the permeability barrier, providing structural support, and determining the cell shape [1–3]. The cell envelope consists of an asymmetric outer membrane (OM) and inner membrane (IM) that are separated by a periplasmic space containing a thin and rigid peptidoglycan layer. Lipoproteins and integral membrane proteins are associated with the IM and the OM. The asymmetry of the OM bilayer primarily results from the location of lipopolysaccharides (LPS) in the outer leaflet and glycerophospholipids (GPLs) in the inner leaflet [4–6]. LPS include lipid A anchored in lipids, O-antigen, and core oligosaccharides [7]. The GPLs with a glycerol backbone, a head group, and fatty acyl chains are mainly phosphatidylethanolamine (PE), phosphatidylglycerol (PG), and cardiolipin (CL) [8]. The asymmetric nature of the OM is maintained by an intermembrane transport system called the Mla (maintenance of outer membrane lipid asymmetry) system, which is composed of six proteins, i.e., the lipoprotein MlaA in the OM, the periplasmic protein MlaC, and the IM complex MlaFEDB [9–12].

The asymmetry of the OM is crucial for the mechanical strength of the envelope. The Mla system is associated with antibiotic susceptibility and intrinsic resistance [13], vesiculation [14–17], pathogenesis [18–21], and virulence [13, 14, 20, 22–25].

**Fig. 1:**
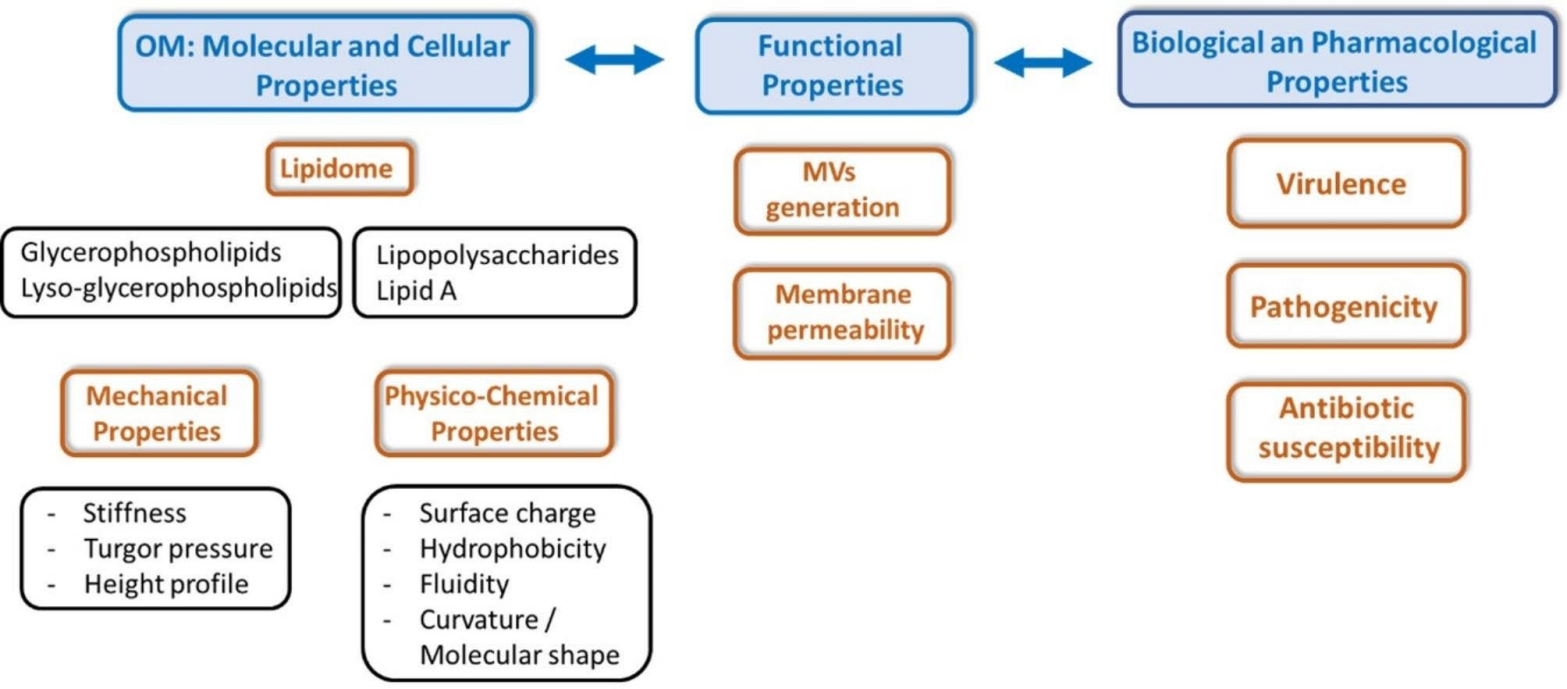
Impact of MlaA on the properties of bacterial OM, with specific insight into molecular, cellular, functional, biological, and pharmacological properties

Significant progress has been made in understanding the transport and assembly of proteins in the OM [26, 27] and the synthesis and transport of LPS [28–30]. In contrast, the transport of GPLs has been uniquely enigmatic [31]. The best-known actor in GPL transport and the maintenance of OM asymmetry is the Mla system. Here, we summarize the information that is available in the literature on the Mla system in general and, more specifically, on the MlaA protein located in the OM. We then explore how MlaA impacts the molecular and cellular properties of the OM with relation to specific functional and biological properties (Fig. 1). Finally, we discuss the potential interest in targeting MlaA in therapy. Of note, throughout the manuscript, the name MlaA was utilized, although VacJ was used in the primary report linked with the intercellular spreading of *Shigella flexneri* [25].

## Maintenance of OM asymmetry: the Mla system

**Fig. 2:**
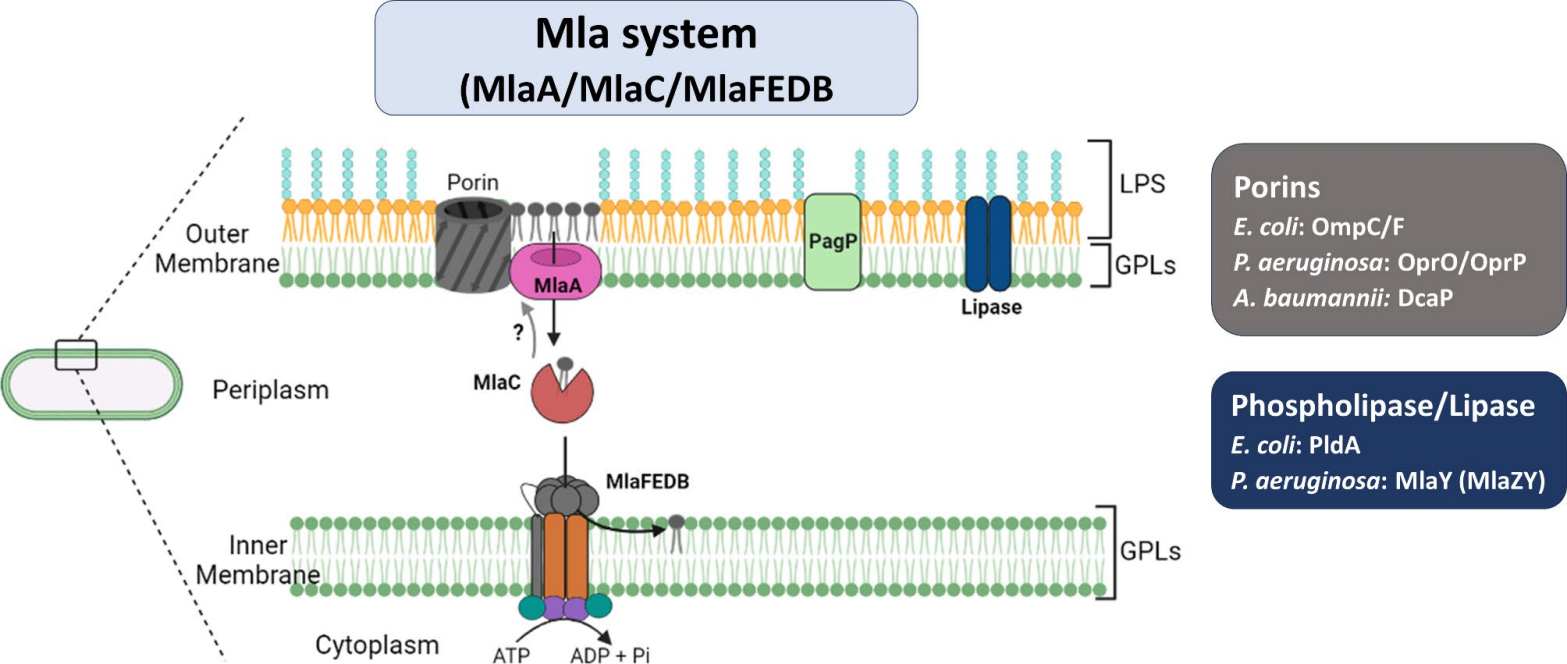
Systems that help maintain lipid asymmetry in Gram-negative bacteria. Mis-localized glycerophospholipids (GPLs) located at the outer leaflet of the OM are transferred by MlaA to a periplasmic protein, MlaC. MlaC further transfers them to the MlaFEDB complex at the IM, which interacts with proteins included porins (OmpC/F in *E. coli* [46, 47]; OprO [48]/OprP [49] in *P. aeruginosa*; and DcaP in *A. baumannii* [50]). Phospholipase (PldA in *E. coli* [42]) and lipase (MlaY in *P. aeruginosa* [51]) may participate in the maintenance of OM asymmetry by managing surface-exposed GPLs (adapted from [52, 53])

The Mla system is a complex of proteins that includes MlaA, MlaC and MlaFEDB (Fig. 2) [9, 32]. MlaA is an OM α-helical membrane protein. MlaA was predicted to be a periplasmic exposed lipoprotein involved in the removal of GPLs from the outer leaflet of the OM that interacted with nonspecific diffusion channels called porins (substrate < 600 Da, e.g., OmpF and OmpC in *Escherichia coli*) to stably position MlaA at the correct depth in the OM, thereby allowing the periplasmic protein to access the outer part of the cell envelope. MlaC is a periplasmic protein [33] and is a predicted substrate-binding protein [34]. The complex MlaFEDB with a stoichiometry of 2:2:6:2 [35] is located at the IM. Within this complex, MlaE and MlaF are the core elements of the ABC transporter. These homodimers function as transmembrane domains (TMDs) and nucleotide-binding domains (NBDs), respectively. The accessory IM protein MlaB, with a sulfate transporter and anti-sigma antagonist (STAS) domain, is bound to MlaF on the cytoplasmic side of the complex and has been implicated in stabilizing the complex and ATP hydrolysis [36, 37]. MlaD has a periplasmic region with a mammalian cell entry (MCE) domain and a transmembrane helix. The MCE domains have been implicated in lipid uptake in Gram-negative bacteria and the retrograde transport of GPLs in chloroplasts [38, 39].

After exposure to various stresses, including divalent cation deprivation, EDTA (ethylenediaminetetraacetic acid), phages, antibiotics, or heat shock [40, 41], Gram-negative bacteria regulate their OM homeostasis by, among other things, disturbing LPS and accumulating GPLs at the outer leaflet of the OM [9, 42]. MlaA removes mislocalized GPLs from the outer leaflet of the OM and transfers them to a periplasmic soluble protein [32], MlaC [43, 44]. The energy released after the hydrolysis of ATP by the MlaFEDB complex is used to extract the GPLs from MlaC and then transfer them to MlaD [9, 12, 36, 45]. MlaD forms a ring-shaped homohexamer that defines a central hydrophobic pore and is thought to allow GPLs to move through [42, 45, 46]. After that, GPLs move into the MlaE cavity and eventually are incorporated into the IM.

The two enzymes PldA and PagP are also involved in maintaining the OM lipid composition. These enzymes utilize GPLs in the outer leaflet of the OM as a substrate. PldA is a phospholipase that generates lysophospholipids and fatty acids. PagP is a palmitoyl transferase that transfers the *sn*-1 palmitate (C16:0) residue of GPLs to lipid A and PG using a recognition site on the outer leaflet [54–58]. PagP activity promotes lipid A acylation and generates lysoglycerophospholipids as a byproduct [55, 56, 59]. PldA and PagP are expressed at low levels and activated under stressed conditions. The primary functions of PldA and PagP differ. PldA [42, 60] is activated under conditions of membrane stress to digest GPLs in the outer leaflet of the OM, as high levels of GPLs in the outer leaflet destabilize OM barrier function. The main function of PagP is modifying LPS through its role as a sensitive in vivo reporter of GPL levels. *pagP* is induced by the PhoP/Q stress response, which senses limited divalent cations [56]. PhoPQ directly regulates the transcription of *pagP* in *Salmonella typhimurium* [61]. In *Klebsiella pneumoniae* ATCC BAA-2146, CrrAB (a two-component regulatory system reported to confer high-level polymyxin resistance and virulence in *K. pneumoniae*) is also a regulator of *pagP* [62]. In *Haemophilus influenzae*, *pagP* and *pldA* homologues are absent despite the fact that the Mla system is present [63, 64]. In the *Neisseria gonorrhoeae* genome, bioinformatics analysis did not identify a *pagP* homologue [14]. How MlaA is related to PldA and PagP is unclear. Malinverni and Silhavy have shown that the function of the Mla pathway is distinct from, but related to, that of PldA. The function of the Mla transport system is not simply to induce or activate PldA [9]. Additionally, *∆mlaA* increases the production of lipid A palmitoylation in *E. coli* in both high and low osmolarity conditions [46]. Deletion of both *mlaA* and *pldA* in *E. coli* further increases lipid A palmitoylation in the stationary phase [9]. Interestingly, in *Pseudomonas aeruginosa*, mislocalized glycerophospholipids are removed from the outer leaflet by PA3239 (renamed MlaZ, a homologue of *E. coli* MlaA), transferred to PA3238 (renamed MlaY), and degraded. MlaA and MlaZ probably have overlapping roles in maintaining the lipid asymmetry of the OM. The MlaYZ system bypasses the need to remove potentially toxic glycerophospholipid degradation products from the outer leaflet by promoting their recycling [51].

If the transport of GPLs across the bacterial cell envelope is fundamental for OM biogenesis and homeostasis, deciphering between retrograde and anterograde GPL trafficking remains an area of active research in this field.

### Retrograde/anterograde transport of glycerophospholipids

In 1977, Jones and Osborn demonstrated that *S. typhimurium* could rapidly translocate GPLs from the OM to the IM [65]. Later, in 2009, the Mla system in *E. coli* was found to help maintain GPL homeostasis in Gram-negative bacteria [9]. The Mla system is thought to be involved in the retrograde trafficking of GPLs; in retrograde trafficking, GPLs are transported from the OM to the IM in an ATP-dependent manner [9, 66] as demonstrated in life-threatening bacteria such as *E. coli* [46], *P. aeruginosa* [20, 24, 52], *Acinetobacter baumannii* [67], *N. gonorrhoeae* [14] and *Bordetella pertussis* [16].

However, based on early biochemical results that showed ATP-independent transfer of GPLs from MlaD to MlaC [68, 69], several groups have suggested that transport could occur in an anterograde fashion (from IM to OM). Mainly, anterograde phospholipid transport has been proposed in *A. baumannii* [70]. However, by comparing the strains used [67], a number of differences became readily apparent, including a strong growth defect in the ∆*mlaF* mutant from the Kamischke et al. study [70]. A link between the growth defect of ∆*mlaF* and a mutation present in *obgE*, which encodes a GTPase involved in the stringent response [71], has been observed [67]. Additional biochemical studies demonstrated that ATP-dependent transport is predominantly retrograde [12], and the hydrolysis of ATP is key to preventing the spontaneous transfer of GPLs from MlaD to MlaC, and thus preventing anterograde transport [66]. Moreover, the directionality of Mla transport could even evolve differently in different groups of bacteria, with some having retrograde transport and others having anterograde (or both) modes of transport. How the collapse of the lipid-binding pocket could lead to the translocation of lipids into the surrounding membrane (retrograde) instead of upwards into the periplasm (anterograde) is still unclear [44]. Part of the debate stems from differences in experimental approaches, the selection of the protagonist (e.g., MlaD per se or included in the complex MlaFEDB), the specific protein or protein complex envisaged, or difficulties in robustly separating the outer and inner membranes [67]. The question of efficiency and potential evolutionary advantage must also be taken into consideration. Structural studies utilizing 3–4 Å resolution approaches, such as cryo-EM studies, might provide new critical information. Therefore, structural and mass spectrometry data on *P. aeruginosa* MlaC revealed that MlaC can carry two diacyl GPLs, such as PE, or one tetra-acylated diphosphatidylglycerol, such as CL [52]. Regarding the MlaFEDB complex, its ability to transfer GPLs to MlaC [68, 69] has been demonstrated, but the MlaA-OmpF/C complex was missing from these experiments. Current functional, structural [12] and biochemical [66, 67] data strongly favour retrograde transport, as was nicely reviewed in [35]. In addition, two recent works support retrograde transport. First, Guest and colleagues demonstrated that the function of the canonical Mla system in *P. aeruginosa* overlaps with a putative lipase that is fundamentally inconsistent with anterograde glycerophospholipid transport [51]. Second, the recent discoveries of AsmA-like proteins [72–74], which invoke a picture analogous to LPS transport by the Lpt complex, deliver phospholipids to the OM in a much more efficient manner than would be possible by anterograde Mla transport.

## MlaA, an OM lipoprotein of the Mla system

### Genetics and phylogenetics

**Fig. 3:**
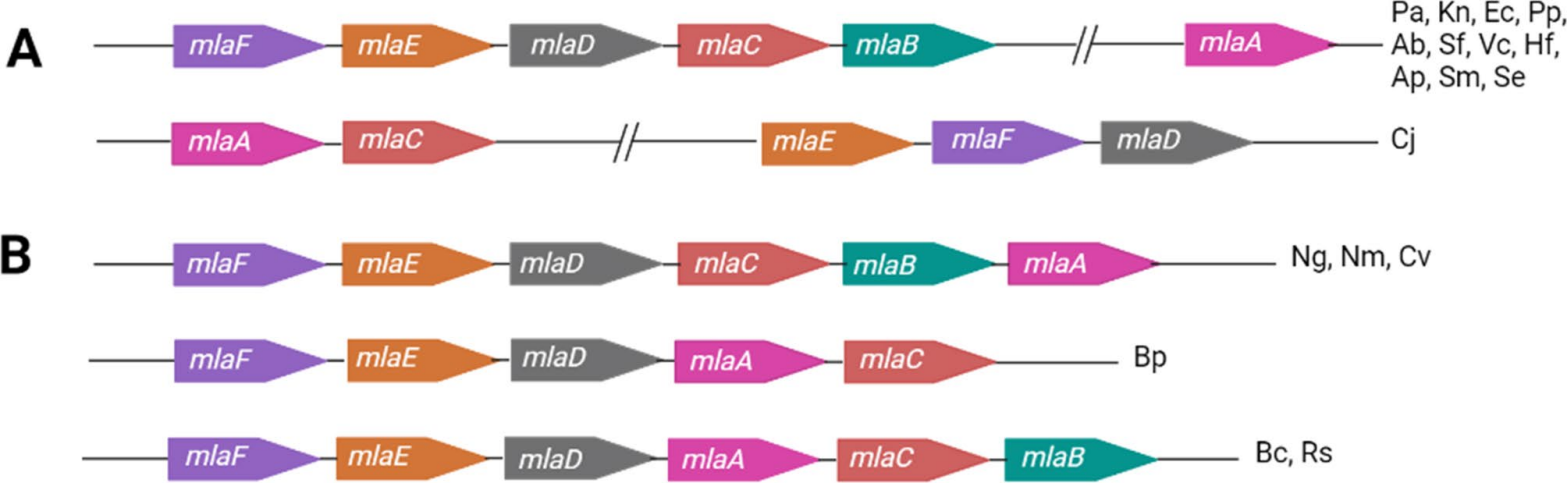
Genetic organization of the Mla system among different bacterial species (A) Discontinuous Mla system, and (B) continuous Mla system. Pa: *P. aeruginosa*, Kn: *K. pneumoniae*, Ec: *E. coli*, Pp: *P. putida*, Ab: *A. baumannii*, Sf: *Shigella flexneri*, Vc: *Vibrio cholera*, Hf: *H. influenzae*, Ap: *A. pleuropneumoniae*, Sm: *S. marcescens*, Se: *S. enterica*, Cj: *C. jejuni*, Ng: *N. gonorrhoea*, Nm: *N. meningitidis*, Cv: *C.violaceum*, Bp: *B. pertussis*, Bc: *B. cepacia*, and Rs: *R. solanacearum* (Adapted from [13, 16]). Genes are not drawn to scale

The genetic organization of the Mla system is diverse among Gram-negative bacteria (Fig. 3). In *P. aeruginosa* [20, 52], *K. pneumoniae* [14], *E. coli* [9, 52], *P. putida* [52, 75], *A. baumannii* [70], *S. flexneri* [22], *Vibrio cholerae* [76], *H. influenzae* [64], *Actinobacillus pleuropneumoniae* [77], *Serratia marcescens* [14] and *Salmonella enterica* [14], the genetic organization is discontinuous; *mlaFEDBF* is clustered as an operon in the genome and *mlaA* is interspersed in the genome. In *Campylobacter jejuni, mlaA* and *mlaC* are clustered together, and *mlaD, mlaE*, and *mlaF* are found together [15]. In *N. gonorrhoeae* [14], *Neisseria meningitidis* [14], and *Chromobacterium violaceum* [78], *mlaA-mlaBCDEF* is clustered as an operon in the genome, suggesting that the regulation of Mla complex expression differs in various bacterial species [14]. In *B. pertussis*, all *mla* genes are found as an operon without *mlaB* [16]. *mlaB* is absent in α and ε proteobacteria [15]. *Burkholderia cepacia* [13] and *Ralstonia solanacearum* [52] also have a continuous operon but with different organizations. The Mla system is highly conserved and prevalent among Gram-negative bacteria [13, 14, 16], actinomycetales and plant chloroplasts [79, 80]. The homologous Mla system is known as Ttg2 (toluene tolerance genes) in *Pseudomonas putida* [75], as VacJ-YrbCDEF in *H. influenzae* and *V. cholerae* [17, 76] and as the Vps-VacJ ABC transporter in *S. flexneri* [22, 25].

Focusing on amino acid sequence analysis, the *mlaA* sequence is highly conserved even when a bacterium is isolated from a different host. Comparative analysis of different strains of *Pasteurella multocida* [81] and *Glaesserella parasuis* [82, 83] show 99.6 to 100% identity in amino acid sequences within species. The retrieved NCBI sequences from Indian strains of *P. multocida* (cattle, buffalo, duck, pig, sheep, quail, rabbit, chicken and turkey) show > 98% amino acid similarity, and proline, aspartic acid, glutamic acid, serine and threonine are the variable amino acids [84]. Similarly, thermophilic *Campylobacter lari* isolates from a wide range of sources (seagull, water, humans, and chicken) show high conservation [85]. Interspecies, *P. multocida* shows between 63.0 and 86.6% amino acid identity with other species of *Pasturella* (*P*. *dagmatis*, *P*. *bettyae* and *P*. *pneumotropica*). Moreover, *Actinobacillus*, *Aggregatibacter*, *Haemophilus*, and *Pasteurella* have 54–98% amino acid identity [81]. When the similarity at the amino acid level in a diverse range of Gram-negative bacteria in comparison to *P. aeruginosa* MlaA was analysed, the percentage identity for two *Pseudomonas* species (*putida* and *syringae*) was approximately 67–69%. For other Gram-negative bacteria (*A. baumannii, N. gonorrhoeae, C. jejuni, Caulobacter crescentus, V. cholerae, H. influenzae, S. flexneri, E. coli, and S. typhimurium*), it decreased and ranged from 32 to 41% [86]. Of note, more *mlaA*-like homologues are found in *Francisella tularensis* [87], *Burkholderia multivorans, Desulfovibrio vulgaris*, and *P. aeruginosa* [51].

By searching for homologues of *P. aeruginosa* MlaA (PA2800) in a curated database containing the proteomes of *Mycobacterium tuberculosis*, *Corynebacteria glutamicum*, and 56 Gram-negative bacteria from diverse phyla using the UniProt BLAST program BLASTP with an E-value threshold of 0.0001 and the BLOSUM62 matrix [88], Guest et al. built the MlaA phylogenetic tree [51]. *P. aeruginosa* MlaA is in a monophyletic group with homologues from *B. multivorans*, *N. meningitidis*, and *B. pertussis* [51]. The homologues from *B. multivorans* are closely related and in the same monophyletic group as *P. aeruginosa* MlaA, but the homologues from *D. vulgaris* are part of different monophyletic groups [51]. Within the phylogenetic tree, SPaseI-cleaved MlaA homologues are clustered separately from those containing a lipoprotein signal peptide (SPaseII-cleaved proteins) [14]. As such, a lipoprotein signal peptide has been reported in *A. pleuropneumoniae* [77, 89], *S. flexneri* [14, 25], *P. multocida* [81], *Xanthomonas citri* subsp. *citri* [90], *E. coli* [14], *H. influenzae* [14], *G. parasuis* [83], *Haemophilus ducreyi* [14], *V. cholerae* [14], *F. tularensis* [87], *S. marcescens* [14], *and Yersinia enterocolitica* [14]. In contrast, no signal peptide was reported in *C. lari* [85] or in *N. gonorrhoea*, *C. jejuni, N. meningitidis*, *Neisseria lactamica, Neisseria weaveri*, *P. aeruginosa*, *P. putida* and *C. crescentus* [14]. The reason for the presence (or absence) of a signal peptide is unclear. One of the potential reasons, but probably not the only one to consider, is the different approaches used (bioinformatic analysis [14, 77, 81, 83, 85] or experimental [25]). The former does not fully consider the complexity of the biological environment, including osmotic pressure [91], the presence of chaperones, dynamic conformational changes, etc.

### mlaA sequence and mutations

Inactivation of any of the *mla* genes has been shown to inactivate the Mla system [9] with multiple consequences, including increased antibiotic resistance (see below). For *mlaA*, numerous mutations have been reported in *E. coli* under tetracycline pressure [92] or after several passages through different concentrations of chlorhexidine; these mutations lead to frame shifts or premature stop codons [93]. In *V. cholerae*, subinhibitory concentrations of polymyxin B caused mutations in genes involved in the maintenance of OM asymmetry (*mlaA* and *mlaF*), the regulation of virulence (*ihfA*), and cell wall synthesis (*dacB*) [94]. Spontaneous colistin-resistant progenies generated from Vietnamese clinical isolates of *A. baumannii* show several mutations, including in *mlaA* [95].

The consequences of *mlaA* mutations in pathogens are diverse. In *N. gonorrhoeae* mutations in *mlaA* induced by exposure to sublethal concentrations of chlorhexidine enhances resistance not only to chlorhexidine but also to azithromycin and ciprofloxacin, which is suspected to be a result of cross-resistance [96]. In *E. coli* [9] and *S. flexneri* [97], mutations within the *vps-mlaA* transporter genes result in increased sensitivity to SDS (sodium dodecyl sulphate). Distinct from the loss-of-function mutations discussed earlier, a dominant mutation in *mlaA* disrupts the lipid balance of the OM by a mechanism involving the activation of phospholipase A, resulting in increased levels of LPS and OM vesiculation that ultimately undermine the integrity of the cell envelope by depleting inner membrane phospholipids [98].

### mlaA expression

The expression of *mlaA* is dependent on the anatomical site of infection [14]. In addition, a decrease in *mlaA* expression is reported (i) after the deletion of the ferric uptake regulator (fur), as observed in distantly related Gram-negative bacteria, including *H. influenzae* RdKW20, *V. cholerae* AC53 *∆fur* and *E. coli BW ∆fur* [17]; (ii) under iron deprivation, as reported in *N. gonorrhoea* [14]; (iii) in the presence of small peptides, such as NPSRQERR, PDENK, and VHTAPK, that are derived from *Lacticaseibacillus rhamnosus* GG and concomitantly decrease *ompC* and *ompF* expression in *E. coli* [99]; and (iv) after incubation with the bile salt sodium taurocholate, which leads to a decrease in the relative gene expression *of mlaA* 2-fold in *C. jejuni* wild-type (WT) strains (11168 and 488) [15]. In contrast, an increase in *mlaA* expression has been reported (i) in the response of *P. putida* KT2440 to phenol [100] and (ii) in *Pasteurellaceae*, where deletion of *qseC*, a sensory histidine kinase of the QseBC two-component system, upregulates the expression of several genes, including *mlaA* [101].

In the clinic, changes at the protein level are often observed. *N. gonorrhoea* strains from geographically distinct areas and different years (clinical isolates from Baltimore, 1991–1994, clinical isolates from Seattle, 2011–2013 and WHO 2016 reference strains) have different MlaA levels, whereas many clinical isolates of non-typeable *H. influenzae* have lower levels of MlaA [14, 63]. Furthermore, in a study comparing an *A. baumannii* colistin-susceptible clinical isolate to its colistin-dependent subpopulation, downregulated expression of proteins involved in the maintenance of OM asymmetry, including MlaA, was observed in the latter isolates [102].

### MlaA structure and MlaA-interacting proteins

If homologous sequences have similar structures and similar functions, inferring functional similarity based solely on significant local similarities is less reliable than inferences based on global similarity and conserved active site residues [103]. Thus, knowledge of the protein structure at the molecular level is critical, especially if the aim is therapeutic targeting.

The crystal structures show that MlaA is a ring-shaped α-helical protein almost entirely embedded into the inner leaflet of the OM [32]. The shuttle-like mechanism to move GPLs from the OM to the IM is critically dependent upon the conformational changes that MlaA might undergo during this process, the putative specificity for one or another GPL, and the interface between MlaA and MlaC. MlaA has a central amphipathic channel whose size is constrained by a helix 6 and a loop [35]. All the α-helices, except helix 6, run parallel to the OM, and as a consequence, they act as a barrier that blocks access to GPLs, thereby preventing their translocation from the inner leaflet to the pore [35]. The MlaA structure explains how it selectively accepts GPLs from the outer leaflet while blocking access to inner leaflet GPLs. The channel has a semicircular ridge with its top end located at the interface of the outer leaflet [35]. Molecular dynamics simulations show that the polar headgroups of GPLs misplaced in the outer leaflet interact with the ridge of MlaA and move downwards into the amphipathic channel, which causes tilting of the acyl chains. This conformational change in MlaA [35] could result from the binding of MlaA to MlaC, as it is known that the function of these proteins might be highly regulated by protein‒protein interactions [44]. Such interactions might induce a displacement of the pore loop and helix 6, thereby exposing the hydrophobic region of the channel wide enough to allocate the acyl chains [35]. The dynamics of the loop appear to control whether MlaA exists in a “closed” or “open” state and thus access to GPLs through the channel. A mutation that likely rigidifies the loop locks MlaA in the nonfunctional closed state, whereas mutations that affect interactions with the loop favour the open state and give rise to exacerbated phenotypes [47]. Thus, one key aspect of MlaA function resides in the hairpin loop structure juxtaposed against the hydrophilic channel.

In *E. coli*, MlaA interacts with OmpC/F, a trimeric porin that typically allows for the passage of hydrophilic solutes across the OM through a hydrophilic channel within the OM-embedded MlaA [32, 46, 47]. *E. coli* lacking OmpC accumulates GPLs in the stationary phase [46]. How OmpC helps maintain OM lipid asymmetry as part of the complex is not clear. Why only MlaA–OmpC is active even though both complexes have virtually identical structures is a matter of concern. One hypothesis is that OmpC may play a passive role and simply be important for stabilizing the structure and orientation of MlaA in the OM [47]. Arg_92_ may somehow affect interactions between OmpC and the MlaA_F133–R205_ peptide, possibly influencing MlaA conformation in the OM and, in turn, properties of the hydrophilic channel [47]. In *K. pneumoniae*, MlaA is copurified with OmpF instead of OmpC [32]. In *P. aeruginosa* and *A. baumannii*, both strains lack OmpF and OmpC porins [35, 104], despite these strains expressing MlaA homologues with structures very similar to that of MlaA of *E. coli* [52]. Other proteins, such as OprO [48] and OprP [49] in *P. aeruginosa* and DcaP [50] in *A. baumannii*, could interact with MlaA, resulting in its stabilization and proper orientation in the OM [47]. However, how MlaA is placed in the OM in the absence of OmpF/C and how other proteins like OprO/P and DcaP could interact with MlaA are unanswered questions.

## Impact of mlaA deletion on OM lipid composition and biophysical properties

If the primary role of MlaA is to transfer mislocalized GPLs on the outer leaflet of the OM to MlaC and then to MlaFEDB located in the IM, deletion of *mlaA* should result in major changes in OM lipid composition when compared to that of WT. In *P. aeruginosa*, total GPL abundance increased with a prominent effect on PE levels. The presence of higher amounts of GPLs in the OM of *P. aeruginosa ∆mlaA* could activate enzymes such as PldA and/or PagP [105], resulting in the production of lyso-derivatives. Interestingly, the profile of total lyso-derivatives was similar to that observed for GPLs, with a tendency to increase in the OM of the *P. aeruginosa ∆mlaA* strain when compared with that of the WT [86]. At the headgroup level, the increase in lyso-derivatives mostly resulted from an increase in LPG compared to LPE. The reasons underlying this change are still unclear but could result (i) from the lack of PldA and PE activity, (ii) the higher stability of the OM when PE is flipped due to its zwitterionic character and small headgroup size [106], of (iii) the increased release of LPE in MVs derived from the *∆mlaA* strain.

ln parallel, compared with the *P. aeruginosa* WT strain, LPS levels in the *ΔmlaA* strain were significantly decreased [86], similar to what was reported for the *∆mlaF A. baumannii* mutant [107]. In contrast with *∆mlaF E. coli* mutant [96]. The reasons for the decrease in LPS in *P. aeruginosa ∆mlaA* compared to WT are unclear and could be linked to a decrease in LPS synthesis resulting from a decrease in LpxC stability [29, 108, 109] or competition for a common substrate for GPLs and LPS synthesis, i.e., β-Hydroxymyristoyl–ACP [110, 111]. Additionally, restricted LPS transport to the OM [110, 112, 113] or the release of LPS from the OM within MVs could be involved.

The decrease in LPS content in *P. aeruginosa ∆mlaA* compared to WT is accompanied by structural modifications of the LPS embedded in lipids (lipid A). These changes in lipid A are driven through the activation of two two-component systems (TCSs) [86], PhoP-PhoQ (PhoPQ) and PmrA-PmrB [114], which results in OM remodelling through lipid A palmitoylation and the addition of 4-amino-4-deoxy-L-arabinose, which could induce changes in virulence [115] and lead to the potent induction of human cytokines [116].

Whether and how these changes in OM lipid composition affect the biophysical and mechanobiological properties of the OM is a growing area of research. These changes may include strengthening the bacterial OM, altering membrane fluidity and surface charge, and increasing membrane curvature [117–120]. Regarding the impact on membrane permeability [121], deletion of *mlaA* did not induce any gross change in membrane permeability [20], despite a higher susceptibility to external compounds [22, 24, 63, 77]. Focusing on the potential impacts on mechanobiological membrane properties, the changes in lipid composition are associated with increased cell stiffness, as was demonstrated in *P. aeruginosa ∆mlaA* [86]. Several hypotheses can be suggested, including (i) longer acyl chains and in turn an increase in the interactions between neighbouring LPS molecules [122–124] and (ii) an increase in the levels of PE or CL compared to that of PG. The relationship between OM lipid composition and OM stiffness might also be more complex, and could involve the presence of peptidoglycan and/or the link between peptidoglycan and the OM [126–127].

## Impact of mlaA on cell phenotypes and membrane vesicles (MV) generation

### mlaA and cell phenotypes

Mutations in *mlaA* may result in changes in the growth rate, cell morphology, and colony morphology. *mlaA* mutants of *N. gonorrhoea* FA1090 [14], *X. citri* subsp. *citri* LJ1516 [90], *P. aeruginosa* PAO1 [20], *H. influenza* Rd KW20 [64], *H. influenza* NTHi375 [64], *E. coli* [23, 98], and *C. jejuni* 11168 [15] show similar growth patterns as their WT isogenic strains. In contrast, *G. parasuis* HS49 [83] and *A. pleuropneumoniae* MD12 *∆mlaA* [77] grow slightly slower than their respective parent strains. Cell morphology remains unaltered in *H. influenza* NTHi375 [64] and *N. gonorrhoea* [14] in the absence of *mlaA* in, as demonstrated in TEM micrographs. In contrast, *G. parasuis* HS49 [83] and *(A) pleuropneumoniae* MD12 *∆mlaA* [77] show altered cell morphology, similar to that of *H. influenza* NTHi375 and Rd KW20, in the presence of bile acid (sodium deoxycholate) [64]. Focusing on the impairment in colony morphotype, *Burkholderia* (*B. dolosa* PC543 and *B. cenocepacia* K56-2) produce smaller colonies on LB agar in the absence of *mlaA*, whereas in *P. aeruginosa* [13], *X. citri subsp. citri* [82] and *E. coli* K-12 [13], no phenotypic differences were visualized.

### mlaA and membrane vesicle (MV) production

A close link between the Mla system and the generation of MV-OM- and/or IM-derived nanosized (10–300 nm in diameter) proteoliposome particles [128, 129] has been reported. For distantly related Gram-negative bacteria such as *E. coli* [23, 130, 131], *P. aeruginosa* [86], *C. violaceum* ATCC12472 [78], *H. influenzae* [17], *V. cholerae* [17, 76], *N. gonorrhoeae* [14], and *C. jejuni* [15], the deletion of *mla* has been associated with an increase in MV generation. The rationale for such a relationship between MV generation and the Mla system likely results from one of the main mechanisms governing MV generation, increased membrane curvature. Three processes play a role in increasing the membrane curvature, (i) the accumulation of GPLs in the outer leaflet of the outer membrane, (ii) modifications of lipid A structures [117] or (iii) the insertion of released chemical signals [called autoinducers] such as acylated homoserine lactones [132–134]. Interestingly, MVs are not produced because of the compromised OM as following the deletion of *mlaA* in *H. influenzae* Rd KW20 [17] and *C. jejuni* [15], the OM integrity largely remained intact. In contrast, overexpression of *pldA* partially reduced MV production in *N. gonorrhoea ∆mlaA* [14], and clinical isolates of *H. influenzae* NTHi have low *mlaA*, correlating with increased MV production [63]. Similarly, the last-resort antibiotics used in the clinics to treat Gram-negative infections further increased the production of MVs in the *N. gonorrhoea ∆mlaA* mutant [14], and sodium taurocholate increased MV production by decreasing *mlaA* and *mlaC* levels in *C. jejuni* 11168 and 488 [15].

MVs of *H. influenzae* Rd KW20 *and V. cholerae* AC53 show no difference in terms of the mean and mode size of MVs [17], whereas *C. violaceum* ATCC 12472 and *C. jejuni* 11168 produce smaller MVs with *∆mlaA* compared to WT [15, 78]. Some changes in lipids were also observed. *H. influenzae* RdKW20 *∆mlaA* has an altered MV lipidome. PE remains the predominant GPL in the OM and MVs of *H. influenzae* RdKW20 WT and *∆mlaA*. Total PE increases in the MVs but not in the OM of *H. influenzae* RdKW20 *∆mlaA* compared to the WT. Moreover, fatty acid composition analysis of MVs revealed a significant decrease in C16:0 [17].

MVs have been found to be pivotal for bacterial physiology, nutrient uptake during starvation, horizontal gene transfer, biofilm formation, host‒pathogen interactions in pathogenesis, virulence, and removing toxic molecules [130, 135–137]. Interestingly, MVs have emerged as a promising tool in vaccinology [138], and MVs, along with MlaA, could be effective immunogens for the prevention of infections (see further). The use of MV-based vaccines as an alternative approach to whole-cell vaccines has been proven to be effective for some Gram-negative bacterium, i.e., *N. meningitidis* [139], and *A. pleuropneumoniae* infections in a serovar-independent manner [89]. However, potential drawbacks must be taken into consideration, including the horizontal transfer of genetic material [140]. If the latter is critical for the evolution of many organisms, this is also a primary mechanism for the spread of antibiotic resistance [141]. The increased generation of MVs induced by *mlaA* deletion, e.g., could overcome the increase in serum sensitivity induced by downregulated the expression of proteins in the Mla transport system [17].

## Role of *mlaA* in virulence and pathogenicity

Gram-negative bacteria produce a large variety of virulence factors that promote bacterial colonization, attachment, and survival. The hallmarks of virulence are biofilm formation, motility, quorum sensing, etc. [142–144]. *mlaA*, also named *vacJ* (virulence-associated chromosome locus J), is required for the intracellular spreading of *S. flexneri* associated with bacterial transmission [25]; furthermore, *mlaA* impacts these virulence-related processes. Deletion of *mlaA* reduces biofilm biomass and thickness as was reported in *A. pleuropneumoniae* MD12 [77], *G. parasuis* [83], *P. aeruginosa* [145], or in the plant pathogen, *X citri* subsp. *citri* [90]. However, other phenotypes have been observed, as in *N. gonorrhoea ∆mlaA*, where there was no effect on biofilm formation [14]. Moreover, in *B. pertussis*, inactivation of *mlaF* alone or together with *pldA* results in increased biofilm formation [16]. Why the response to *mlaA* deletion varies in different strains is unclear, but biofilms could be only one part of the collective stress response [146].

MlaA also influences the secretion of virulence factors in numerous Gram-negative bacteria [14, 20, 23, 24]. Flagella- and type IV pilus-mediated motility contribute to virulence in numerous bacteria, including *P. aeruginosa* [147]. In *P. aeruginosa*, deletion of *mlaA* reduces swimming, swarming, and twitching [145]. *X. citri* subsp. *citri ∆mlaA* also decreases swarming motility on semisolid agar [90]. Using type IV pili, *P. aeruginosa* actively measures substrate stiffness and uses this information to regulate virulence-related genes when cells are in a specific stiffness range [148].

Moving on to pathogenicity, the effects induced by MlaA are dependent upon the strain and the animal model [82]. A transposon insertion mutation in *A. baumannii* resulted in 15-fold attenuation in a mouse pneumonia model [21]. Loss of *mlaA* in *P. aeruginosa* PAO1 led to 40% survival after 3 days of lung infection by haemorrhage minimalization and reduced survival of *P. aeruginosa ∆mlaA*, which led to less production of disseminated inflammation lesions [20]. Deletion of *mlaA* in *X. citri subsp. citri* significantly reduced the growth and population on mandarin orange leaves, with the *ΔmlaA* strain inducing fewer canker lesions on mandarin orange plants 14 days post incubation [90]. *F. tularensis ∆mlaA*-challenged BALB/c mice led to less than 50% survival. Absence of *mlaA* increases the cytotoxicity of *E. coli* in silkworm. A double mutant of *mlaA* and *pldA* decreased the killing of silkworm [23]. A *Drosophila* feeding assay showed increased mortality in *Drosophila melanogaster* following *P. aeruginosa* PAO1 *∆mlaA* infection compared to infection with the WT [24].

Interestingly, the expression of *mlaA* genes is positively correlated with serum resistance among clinical isolates. *H. influenzae* NTHi adapts to inflammation in the lower respiratory tract by increasing the expression of *mla* genes to minimize recognition by bactericidal anti-oligosaccharide antibodies [63]. Loss of serum resistance in *mlaA* mutants was correlated with increased binding of natural immunoglobulin M in serum, as well increased binding to anti-oligosaccharide monoclonal antibody. Similar observations were made in *G. parasuis* [83] and in *A. pleuropneumoniae* MD12 [77].

## MlaA, a candidate for improving the effect of antibiotics?

In the literature, the Mla system has been proposed as a novel drug target because of its roles in resistance to host imposed stress and antibiotics [70, 99].

*mlaA* deletion induced an increase in susceptibility to antimicrobials, as reported for peptides LL-37 and murine cathelicidine-related antimicrobial peptide (CRAMP) in *P. aeruginosa* PAO1 [20], polymyxin B in *N. gonorrhoea* [14], colistin (polymyxin E) in *P. aeruginosa* [20], and *H. influenzae* [64]. An increase in susceptibility was also reported for multiple antibiotics, including macrolides, tetracyclines, fluoroquinolones, chloramphenicol, and rifampin, in the *mla* mutants of *B. cenocepacia and B. dolosa* [13], *A. pleuropneumoniae* [77], *P. aeruginosa* PAO1 [13, 24], *E. coli* [23], *G. parasuis* [83], *and H. influenzae* [64]. Similarly, increased MlaA levels in *S. enterica serovar Typhimurium* R200 result in increased resistance to ceftriaxone, and deletion of the *stm3031* (putative OM protein) gene returns the MlaA levels to normal [149]. Less often, *∆mlaA* is responsible for a decrease in susceptibility to antimicrobials, as was reported for arenicin-3 and vancomycin in *E. coli* [23], polymyxin B in *C. jejuni* [15], and ceftriaxone in *N. gonorrhoeae* [150, 151].

Similar to what is observed for antimicrobials, *mlaA* deletion increased sensitivity to bile salts in *E. coli* [98], *H. influenzae* [64] and *N. gonorrhoea* [14], exogenous free fatty acids in *H. influenzae* NTHi375 and RdKW20 [64], and a combination of SDS and EDTA in *G. parasuis* [83], *A. pleuropneumoniae* [77], *P. aeruginosa* [20], *S. flexneri* [22], and *E. coli* [9] and cetylammonium bromide in *E. coli* [152]. Again, this seems to be highly dependent upon the bacterial strain and the compound since an increase in resistance against cationic antiseptics, such as chlorhexidine, has been reported in *E. coli* [23]. Additionally, deletion of *mlaA* increases sensitivity to osmotic stress in *A. pleuropneumoniae* MD12 [77] and *G. parasuis* [83] and to oxidative stress in *G. parasuis* [83], *C. jejuni* [153], and *N. gonorrhoea* FA 1090 [154]. In contrast, no effect is observed in terms of either oxidative stress in *A. pleuropneumoniae* MD12 [77] or thermal stress in *G. parasuis* [83] and *A. pleuropneumoniae* MD12 [77].

How MlaA might modulate susceptibility to antibiotics is unclear, but the following three noncontradictory hypotheses have been proposed: (i) changes in the physicochemical parameters of the OM and increases in OM permeability [20], (ii) modification in lipid A structure or (iii) the expression of diverse proteins that interact with Mla proteins [13]. First, experimental data do not support a global loss of membrane integrity. In *C. jejuni*, 11168 *∆mlaA* showed no change in membrane stability, as assessed by a membrane potential probe and 3,3’-dipropylthiadicarbocyanine iodide (DiSC3) accumulation [15]. Likewise, no difference in the uptake of NPN (1-*N*-phenyl naphthylamine), a hydrophobic probe used as an indicator of OM permeability in *P. aeruginosa* PAO1 [20], was shown. Second, specific structural changes in LPS, which in turn might affect antibiotic diffusion through the OM, might be critical. Structural variations in LPS and the composition of LPS in the OM can affect certain characteristics such as thickness and modifications in the lipid A portion altering the hydrophobicity of the bilayer; these changes may in turn prevent the permeation of hydrophobic compounds [155]. Thus, exploring the use of MlaA to increase permeability [156, 157] might be a promising means for overcoming resistance to certain antimicrobials. Third, proteins, such as YadH in *E. coli* [158] or BCAL0307 and BCAL0308 in *Burkholderia* [13], whose genes are located downstream of *mla* genes, might form a complex with MlaA. All these changes could be responsible for the remodelling of bacterial OM. Under selection, in an environment where antibiotics are present, remodelling the OM to become drug-resistant would have a competitive advantage when compared to drug-sensitive bacteria.

Genetic studies suggest that the Mla pathway may be a target to potentiate current antibiotics. Focusing on *Burkholderia*, the Mla pathway is present in isolates and is genetically similar in more than 675 *Burkholderia* sequenced strains, supporting the interest in exploiting this pathway. In particular, targeting the Mla pathway with an antibiotic adjuvant in combination with selected antibiotics might be effective against multidrug-resistant pathogens [13]. If the aim is to target MlaA, a selective strategy would be to act at the transcription or posttranscription level as we do not know the mechanisms involved in trafficking of MlaA (kinetic and thermodynamic parameters related to integration into the OM and degradation processes). Taking advantage of the fact that *mlaA* per se is not an essential gene, silencing *mlaA* could resensitize bacteria to antibiotic treatment without imposing its own fitness cost [159]. However, the interest (or not) in targeting nonessential genes remains open for discussion. In this line, processes whose inactivation most directly blocks replication are also attractive antibiotic targets, since even partial inhibition could limit growth [160].

## MlaA, an option for vaccines?

Since OM proteins and lipoproteins play key roles in the interactions of pathogens with the host environment and in the host immune response to infection, a rising question is the interest in utilizing MlaA to improve immunogenicity. Being exposed on the bacterial surface is one of the main criteria for an ideal vaccine antigen candidate [161]. However, since MlaA has been predicted to be a periplasm-exposed lipoprotein [35, 44], this raises the question of how a periplasmic protein can gain access to the outside of the cell envelope. Using an experimental approach, Suzuki et al. (1994) suggested that MlaA (*S. flexneri*) would be exposed on the bacterial surface [25]. Such a discrepancy between prediction studies and experimental studies is probably due to the complexity of the biological environment. In addition, the membrane remodelling observed during the formation of outer membrane vesicles could be responsible for the localization of the protein at the surface. In the literature, several studies have reported that MlaA is a vaccine candidate, including in vaccines against *A. pleuropneumoniae* [65], *N. gonorrhoeae* [14], *P. multocida* [81, 162], *G. parasuis* [82, 83], and *Vibrio parahaemolyticus* [163].

In *A. pleuropneumoniae*, the MlaA protein elicits higher IgG in uninfected pig serum [89], but immunization of pigs with MlaA neither changes the extent of the pathological score of lesions by *A. pleuropneumoniae* [138] nor combats *G. parasuis* disease [82]. Inoculation of *P. multocida* recombinant (r) MlaA gives 33% protection, whereas inoculation of combined rMlaA and two OM lipoproteins (rPlpE + rOmpH) provides 100% protection [162]. Last, MlaA-immunized guinea pigs produced low antibody levels, even though the percentage of cells killed by MlaA was the highest in the whole blood killing assay, as compared to the other OMPs (CsgG, HAPS_0742, Omp26, PlpD, PlpA, and YfeA) tested in the study [164].

If MVs, along with MlaA, could be effective immunogens, their potential endotoxicity must be taken into consideration. LPS, particularly its lipid A moiety, is responsible for the endotoxicity associated with infections by Gram-negative bacteria. Lipid A is recognized by a receptor on innate immune cells that consists of Toll-like receptor 4 (TLR4) and myeloid differentiation factor 2 (MD-2), which triggers the production of proinflammatory cytokines, such as TNFα and IL-1β [165]. The endotoxic reaction elicited by LPS is one of the main reasons for the adverse reactions evoked by whole-cell vaccines against various gram-negative bacteria, including *B. pertussis* [166]. Palmitoylated lipid A confers resistance against the host immune system by interfering with Toll-like receptor 4 in *S. typhimurium* and *P. aeruginosa* [165, 167]. Interestingly, a reduction in the total amount of LPS might be an alternative approach to maintain appropriate endotoxin levels. This approach has been explored by Perez-Ortega and coll. who isolated MVs from LpxC-depleted cells and demonstrated reduced activation of Toll-like receptors [166]. Moreover, the genetic engineering of the lipid A structure to weaken its interaction with TLR4 [120, 168] may also be pursued, as was done in *B. pertussis* [169, 170].

## Concluding remarks and future outlooks

Although the overall architecture, composition, properties, and biogenesis of the cell envelope are conserved among Gram-negative bacteria, there is a great deal of diversity in the details. The OM of Gram-negative bacteria is asymmetric. The Mla system in charge of maintaining OM asymmetry is highly conserved among genetically distant bacterial species. Here, we envisioned MlaA, a protein of the Mla system located in the inner leaflet of the bacterial OM, and explored the downstream effects of *mlaA* deletion. Specifically, we analysed changes in the composition in lipids of the bacterial OM with the consequences on the mechanical and biophysical membrane properties. We also analysed how these changes may result in alterations in the functional (MV generation and membrane permeability) and biological (virulence, pathogenicity, and antibiotic susceptibility) properties of the OM. How MlaA might be targeted to fine-tune bacterial-host interactions is still an open question.

To resolve the controversy in which some groups dispute the widely accepted directionality of phospholipid transport via Mla, it may be interesting to explore how the Mla system works in the phylum *Bacteroidota* (formerly Bacteroidetes) in the near future. Regarding retrograde transport, a more efficient system to remove misplaced outer leaflet GPLs should offer an evolutionary advantage to a bacterium, such as *Bacteroides thetaiotaomicron (B. theta)*, that is in constant contact with bile acids [35]. Regarding anterograde transport, the efficiency argument could also be put forward. The genome of *B. theta* does not include homologues to *mlaA* or *mlaC*, but it has an extended MlaD. This extended MlaD protein could accommodate the acyl chains of the GPLs inside the channel and the polar heads facing the periplasm. This transport would be more efficient than using a cargo protein such as MlaC, potentially favouring anterograde transport. Complementary information could be provided by studying the role of the MlaYZ (in *P. aeruginosa*) and AsmA-like proteins. The rate of synthesis, transformation and breakdown of each phospholipid may also be critical to understanding how OM asymmetry is maintained.

In conclusion, MlaA is an intriguing protein that has multiple functions from helping maintain OM asymmetry to the intracellular spread of bacteria in epithelial cells. We are at the beginning of an exciting journey to decipher the potential of MlaA in fine-tuning the ability to colonize the host by modulating the levels of virulence factors, cell envelope properties, and vesiculation. Further cellular and molecular studies on MlaA and the Mla system are needed to evaluate the opportunity to target proteins in the Mla system, with the aim of fighting the rapid rate of emerging Gram-negative bacterial resistance.

## Data Availability

The data that support the findings of this study are available from the corresponding author upon reasonable request.

## Declarations

### Additional information

No dual publication.
